# The Development of a Screening Questionnaire for Obstructive Sleep Apnea in Children with Down Syndrome

**DOI:** 10.3389/fpsyt.2015.00147

**Published:** 2015-10-20

**Authors:** Emma Sanders, Catherine Mary Hill, Hazel Jean Evans, Catherine Tuffrey

**Affiliations:** ^1^Faculty of Medicine, University of Southampton, Southampton, UK; ^2^Southampton Children’s Hospital, Southampton, UK; ^3^Department of Child Health, Solent NHS Trust, Southampton, UK

**Keywords:** obstructive sleep apnea, Down syndrome, sleep disorders, pediatrics, screening, measurement

## Abstract

Obstructive sleep apnea is a condition which affects an estimated 50% of children with Down syndrome, particularly in their early years. It can cause serious sequelae in affected children but may not be recognized by parents or health professionals. Routine screening has been recommended in some countries, but is not standard practice. There are no validated questionnaire-based tools available to screen this population of children for this particular sleep-related disorder. Using existing validated sleep questionnaire items, we have developed a questionnaire to screen children with Down syndrome up to 6 years of age for obstructive sleep apnea, which corresponds with the recommendations made in UK national guidelines. This paper describes these first steps in demonstrating content validity for a new questionnaire, which will be subject to further in-depth psychometric analysis. Relevance, clarity, and age appropriateness were rated for 33 items using a content review questionnaire by a group of 18 health professionals with expertise in respiratory pediatrics, neurodevelopmental pediatrics, and sleep physiology. The content validity index was calculated for individual items and contributed to decisions about item inclusion. Scale level content validity index for the modified questionnaire of 14 items was at an accepted level of 0.78. Two parents of children with Down syndrome took part in cognitive interviews after completing the modified questionnaire. We describe the development of this 14 item questionnaire to screen for OSA in children with DS from infancy to 6 years.

## Introduction

Obstructive sleep apnea (OSA) is characterized by repetitive partial (hypopnea) or complete (apnea) airway collapse during sleep despite continued respiratory effort. It is believed to affect over 50% of children with Down syndrome (DS) compared to around 1–3% of typically developing children (1–3). Causation in DS is multi-factorial with hypotonia, obesity and craniofacial anatomy all contributing to collapse of a narrow airway, further exacerbated in the pre-school years by growth of the adenotonsillar tissue. Children with DS are at increased risk of adverse complications of OSA (4, 5). Up to 60% of children with DS have congenital heart disease putting them at increased risk of developing pulmonary hypertension (4). Cognitive and behavioral sequelae of OSA seen in typically developing children, namely inattention, reduced academic performance, and daytime behavioral problems (6, 7) are likely to be more problematic in children with DS who have reduced cognitive reserve (8). Treatment options, such as adenotonsillectomy and overnight non-invasive ventilation, can reverse both physical and neurocognitive complications of OSA in typically developing children (9), although there have been no large randomized trials of such approaches in children with DS (10).

Clinical history and examination are poor at predicting OSA in both typically developing children (11–14) and children with DS. Furthermore, parents may not report OSA symptoms in their children with DS (15, 16). This may reflect lack of awareness of nocturnal symptoms on the part of parents, or failure to recognize their significance. Clinical diagnosis alone therefore has limited utility and screening is recommended. Guidelines for screening for OSA in DS vary across the world. The American Academy of Paediatrics recommends referring all children with DS for a sleep study or polysomnography by the age of 4 years (17). The UK Royal College of Paediatrics and Child Health (RCPCH) recommend annual screening of children with DS from infancy until 3–5 years old, with a minimum of pulse oximetry (18). Follow-up cardiorespiratory polygraphy studies are recommended in children with abnormalities on oximetry screening, or where clinical assessment suggests a false negative screening test (18). In effect therefore, the majority of children may require as a minimum cardiorespiratory polygraphy. The impracticality and limited availability of such technology in some parts of the world suggests a need to investigate the validity and utility of simpler screening methods.

There are few screening tools designed for clinical populations of children with developmental disorders, who may differ in their presenting symptomatology. Existing pediatric sleep questionnaires have not been designed or validated for children with DS (19). Their utility in screening for OSA in children with DS is therefore unknown. Furthermore, existing OSA screening questionnaires contain a number of behavioral questions that children with learning disabilities may score positively, even in the absence of OSA. For example, the Pediatric Sleep Questionnaire (PSQ) contains a subscale that asks questions about reduced growth rate and weight gain (20), which are common problems in children with DS that may exist independent of OSA. A questionnaire designed specifically for children with DS should have greater sensitivity and specificity and may provide a useful adjunct assessment to clinical history in screening for OSA (19).

The aim of this study was to undertake the first phase of the development of a questionnaire to be used specifically as a screening tool for OSA in children with DS aged between 6 months and 6 years old. This age range was selected as it includes the screening period recommended by the UK RCPCH (18). Here, we report the methods used and initial evidence for content validity.

## Materials and Methods

This study was approved by the Faculty of Medicine research ethics committee at the University of Southampton.

Prior to testing the psychometric properties of a questionnaire, key stages of development must be completed. A detailed description of the steps required was outlined by Spruyt and Gozal in their paper on the development of pediatric sleep questionnaires (21), as well as by other authors (22). The initial steps are important in providing evidence of content validity, that is, the extent to which the questionnaire measures the intended construct and is appropriate for its intended use. The stages we used are described below.

### Item Generation

Existing pediatric sleep questionnaires, which included questions focusing on OSA were reviewed by the study steering group (Catherine Tuffrey, Catherine Hill, and Hazel Evans) to identify a range of questions, which relate to symptoms relevant to identifying OSA in children with DS. Questions were selected from five existing pediatric sleep questionnaires identified through the literature review, namely the Pediatric Sleep Related Breathing Disorder Questionnaire (PSQ) (20), OSA disease specific quality of life questionnaire (OSA-18) (23), Sleep Disorders Inventory for Students-Children’s form (SDIS-C) (24), Gozal’s adaptation of Brouilettes Questionnaire (25), and the Hong Kong children’s sleep questionnaire (HK-CSQ) (26). These questionnaires have all been previously validated against objective measures, such as PSG (19). Question stems were modified to fit with the chosen response format described below. In addition, the study group devised four new questions to cover symptoms, which were not covered in existing questionnaires.

### Item Response Options

Existing questionnaires were examined by the study steering group and the response format was selected that would be appropriate for all questions and that would relate to a period of time, which parents would be able to most accurately recall. A Likert response format was chosen to rate the frequency of symptoms over a week. The response format chosen was a modification of Gozal’s adaptation of Brouillette’s questionnaire (25) [Never (never in the past 6 months), Rarely (once a week or less), Occasionally (2 times a week), Frequently (three to four times a week), Almost always (more than four times a week)]. The response format was chosen to avoid forcing the respondent into choice of category. An “unsure” option was added to help avoid non-response. The addition of an “unsure” option can, however, introduce some challenges to the interpretation and scoring of responses. The response format was later reviewed for suitability as part of the expert content review and through cognitive interviews with parents.

### Expert Review

Instrument development experts support the use of knowledgeable expertise to objectively measure content validity. For this to be effective, content experts must understand their expert role, the concepts being explored and the intended use of the instrument. A covering instruction sheet was included outlining the process of content review as recommended by Lynn (27). We aimed to recruit 10–20 experts from a range of relevant backgrounds to review the questionnaire for relevance, clarity, and age appropriateness. The literature suggests a minimum of 5 and a maximum of 10–20 experts for content review (22, 28). To allow for non-responders, more experts were contacted than were needed. Ninety experts from the UK Down syndrome Medical Interest Group, a network of health professionals with a specialist interest in Down syndrome, and the UK Sleep Videoconference network, an informal network of pediatric sleep centers in the UK who meet quarterly by video-conference, were emailed individually with details of the project, the content review questionnaire, and instructions for completion. No reward was offered for completion.

Expert opinion was collated using a structured feedback form in the form of a content review questionnaire (22). Questions were arranged into symptom subgroups under the headings; snoring, breathing difficulties, mouth breathing, upper respiratory tract infections, sleep position, restless sleep and frequent awakening, and daytime behavior. Of the 33 items included in the content review questionnaire, 3 items corresponded to snoring, 8 items to breathing difficulties, 5 items to mouth breathing, 4 items to upper respiratory tract infections, 2 items to sleep position, 6 items to restless sleep and frequent awakening, and 5 items to daytime behavior. No indication was given as to the source questionnaire of each item in order to minimize bias. For the purpose of content review, there was intentional over inclusion of questions so that experts could decide which phrasing or symptom construct was most appropriate. Experts were asked to rate the relevance of each item on a four-point scale, where 1 indicated “not relevant” and 4 indicated “relevant.” In addition, experts were asked whether the question was clear (yes or no) and age appropriate (yes or no). Free text comments were invited for each item. Further questions were asked concerning the overall comprehensiveness of the questionnaire and the suitability of the response options.

The content validity index (CVI) was calculated for each item and for the questionnaire as a whole (28). CVI is defined as the proportion of experts who rated a question relevant and is an index of inter-rater agreement. To calculate the CVI for each item, the number of experts who rated the item 3 or 4 (3 = the item needs minor revisions to be relevant, 4 = the item is relevant) is divided by the total number of experts (28).

The CVI can be calculated for individual items (I-CVI) and for the scale as a whole (S-CVI). For this latter measure, we have used the version of S-CVI where the mean of the I-CVI’s is calculated. Where a new question was suggested following review, as this would not have a CVI, this item was excluded from the S-CVI calculation. The literature recommends a CVI value of 0.78 for a question to be deemed as relevant (28). Items rated lower than this were considered for revision or deletion. It is important to understand that the CVI is only one consideration when deciding on the fate of an item and other factors such as content coverage also need to be considered. CVI does not adjust for chance agreement, but as the number of experts increase, the risk of chance agreement decreases (28).

Clarity and appropriateness were rated with a yes/no response followed by free text comments and suggestions. Proportions of experts rating an item positively for clarity and relevance were recorded.

Finally, free text comments aided the selection, rephrasing, and elimination of questions.

### Cognitive Interviews

Cognitive interviews are a recommended method of pre-testing or evaluating questionnaires and focus on the cognitive processes of the respondent in answering questions (29). A sample similar to the final user population is recommended and we therefore interviewed parents of children with DS aged <7 years old. Parents were invited to participate via a local DS parent support group. Cognitive interviews were conducted individually using “Thinking aloud” and “Probing” techniques (29). Parent volunteers were asked to read the items selected for inclusion in the questionnaire and comment as they answered each question. “Thinking aloud” has the benefit of being free from interviewer-based bias and it allows the subject to elaborate on their thought processes. “Thinking aloud,” however, suits some personality types more than others; some subjects may struggle to elaborate on their thought processes, while others may deviate from the subject topic in their answers. “Probes” allow the interviewer to focus the interview around areas of potential response error and prompt the subject into giving their opinions. Examples of probes are; “How did you get the answer of …. nights a week?” and “What does the phrase ‘restless sleep’ mean to you?” Probes can focus the respondent to think about their understanding of the question, recall of information, and degree of estimation needed to answer the question. Use of “probes” has the potential for bias therefore care to avoid leading questions should be taken (21, 29). Parents were asked additional questions concerning; the response format, time frame, clarity of questions, and general layout. Parents were also asked if their child had been investigated for, or diagnosed with, OSA. Comments received from parents during this phase guided the final phrasing of questions, questionnaire completion instructions, and changes to the layout. Ideally, sampling should continue to data saturation, however, the time constraints of this study only permitted one round of interviews. No financial incentives were offered to parents to participate.

### Readability

Following expert review and cognitive interviews, selected and modified questions were subject to the Flesch Reading Ease test (30). This analyses the number of syllables per word and number of words per sentence to produce a readability score. A score of 60–70 equates to a reading age of 13- to 15-year-old students.

## Results

### Expert Review

#### Content Review Questionnaire

Eighteen experts from a range of healthcare backgrounds participated in the content review stage of questionnaire development. The majority (*n* = 10) of respondents were respiratory pediatricians; the others were two neurodevelopmental pediatricians, two sleep physiologists, two specialist nurses, a respiratory physician and a neurophysiologist.

#### Content Validity Index

The CVIs calculated for each item (*n* = 33) ranged from 0.43 to 0.94. Eight items scored a CVI greater than the accepted level of 0.78. The breathing difficulties questions had the highest CVIs, while the upper respiratory tract infections subgroup had the lowest. Full details are given in Table 1.

**Table 1:** **Content review results**.

Item number	Questions	I-CVI	Clarity (%)	Relevance to age group (6 months-6 years) (%)
**Section 1: snoring**				
1	How often does your child snore when they do not have a cold?^a^	0.94	86.67	100
2	How loud is the snore? (*mildly quiet* = *in bedroom; medium loud* = *outside bedroom door; Very loud* = *between floors of the house*)^a^	0.69	80.00	75.00
3	How often can you hear the child snoring from outside of the bedroom door?^b^	0.69	84.62	83.33
**Section 2: breathing difficulties**				
4	Does your child stop or pause breathing during sleep?^a^	0.75	68.75	93.75
5	Does your child struggle to breathe while asleep?^a^	0.88	76.47	93.75
6	If your child struggles to breathe, does their chest suck in?^b^	0.71	56.25	86.67
7	Do you ever shake your child to make him/her breathe again when asleep?^a^	0.71	70.59	76.47
8	Are you ever concerned about your child’s breathing during sleep?^a^	0.65	76.92	82.35
9	How often does your child have breath holding or pauses during sleep?^c^	0.71	78.57	85.71
10	How often has your child made choking or gasping sounds while asleep?^d^	0.86	76.92	100
11	How often is there a period of silence in your child’s breathing followed by a gasp?^b^	0.87	69.23	100
**Section 3: mouth breathing**				
12	Does your child tend to breathe through the mouth during the day?^e^	0.79	75.00	80.00
13	Is your child a daytime mouth breather?^a^	0.69	58.33	58.33
14	How often does your child have mouth breathing during sleep?^c^	0.71	76.92	75.00
15	On waking, does your child have a dry mouth?^e^	0.60	69.23	41.26
16	On waking, is your child thirsty?^b^	0.43	60.00	50.00
**Section 4: upper respiratory tract infections**				
17	How often has your child had colds or upper respiratory infections that affect their breathing at night?^d^	0.46	53.87	66.67
18	How often has your child had a runny nose?^d^	0.50	75.00	81.82
19	How often has your child had difficulty swallowing?^e^ (in comparison to children of the same age)	0.46	66.67	66.67
20	Does your child have a persistent runny nose?^d^	0.42	63.64	70.00
**Section 5: sleep position**				
21	How often does your child sleep in strange positions such as cocking the head backwards or sleeping while sitting upright on pillows or kneeling?^f^	0.73	85.71	76.93
22	Does your child tend to sleep lying on their front?^c^	0.53	92.31	38.46
**Section 6: restless sleep and frequent awakening**				
23	How often does your child have restless sleep?^d^	0.71	64.29	66.67
24	Compared to children of a similar age, how often does your child have frequent awakening?^d^	0.60	57.14	64.29
25	Does your child roll or move around the bed while sleeping?^f^	0.67	92.86	58.33
26	Does your child sweat a lot while asleep?^f^	0.79	84.62	63.64
27	Does your child wake up during night? (More than a child of a similar age)^f^	0.73	92.86	69.23
28	How often has your child had difficulty waking up in the morning?^d^	0.73	78.53	53.85
**Section 7: daytime behavior**				
29	Is your child unusually sleepy during the daytime?^a^	0.86	62.29	50.00
30	Does your child appear sleepy more often in the daytime than children of the same age?^f^	0.73	61.54	53.85
31	Does your child appear to be “on the go” or often acts as if “driven by a motor”?^e^	0.60	72.73	27.27
32	Does your child appear to be more hyperactive than children of a similar age?^b^	0.79	91.67	27.27
33	Has your child stopped growing at a normal rate since birth?^e^	0.47	50.00	66.67

*Items derived from ^a^Gozal’s adaptation of Brouilette’s questionnaire; ^b^New item; ^c^HK-CSQ; ^d^OSA-18; ^e^PSQ; ^f^SDIS-C*.

From the 33 items in the content review questionnaire (see Table 1), 13 items were included in the final selection with 1 further question added; 2 items corresponded to snoring, 3 items to breathing difficulties, 1 item to mouth breathing, 1 item to sleep position, 4 existing items, and 2 items to daytime behavior. Following comments from experts on clarity and content of item 28 on morning wakening, an additional item was written. No items from the upper respiratory tract infection subgroup were selected. Six questions with CVI scores <0.78 (0.69–0.73) were included to ensure the questionnaire had adequate content coverage prior to testing of its psychometric properties in a clinical sample. These questions were revised from their original format according to experts’ comments and are shown in Table 2. The CVIs for included items ranged between 0.69 and 0.94 and the S-CVI for the 13 items where a CVI could be calculated is 0.78.

**Table 2:** **Modifications made to questions selected**.

Original item (number)	CVI score	Modifications	Final version
How often does your child snore when they do not have a cold? (1)	0.94	Use capital letters for “NOT” due to comments that it takes concentration to read “do not have”	How often does your child snore when they do NOT have a cold?
How often can you hear the child snoring from outside of the bedroom door? (3)	0.69	Deleted “door” as experts highlighted the problems with interpretation of open or closed door	How often can you hear the child snoring from outside of the bedroom?
Does your child struggle to breathe while asleep? (5)	0.88	Changed “does” to “how often” to fit with the response format	How often does your child struggle to breathe while asleep?
Do you ever shake your child to make him/her breathe again when asleep? (7)	0.71	Felt that “struggling to breathe” and “needing to be shaken” were good discriminatory questions for OSA but thought that the word “shake” has associations with non-accidental injury therefore parents may be reluctant to answer this question. Changed “shake” to “nudge or touch”	How often do you nudge/touch your child to make them breathe again when asleep?
How often is there a period of silence in your child’s breathing followed by a gasp? (11)	0.87	Changed “period of silence” to “breathing go quiet” as is more descriptive and easier to understand	How often does your child’s breathing go quiet and then he/she gasps?
Does your child tend to breathe through the mouth during the day? (12)	0.79	Changed “does” to “how often” to fit with the response format. Experts highlighted that it may be difficult for parents to identify this and that many children with DS tend to keep their mouth slightly open due to a large tongue volume. Thought however that it was necessary to include one mouth-breathing question	How often does your child tend to breathe through their mouth during the day?
How often does your child sleep in strange positions such as cocking the head backwards or sleeping while sitting upright on pillows or kneeling? (21)	0.73	Changed “cocking” to “tilting.” There was a split of opinion of experts over this question, however the majority felt that sleep position was relevant. This question may not be appropriate for the entire age range as younger children may not be mobile enough to change position	How often does your child sleep in strange positions such as tilting the head backwards or sleeping while sitting upright on pillows or kneeling?
How often does your child have restless sleep? (23)	0.71	Felt that this was a useful clinical question but may not be very specific for OSA as there are many causes for restlessness. This question however had the best expert consensus of the questions aimed at identifying restlessness	How often does your child have restless sleep?
Does your child sweat a lot while asleep? (26)	0.79	Omitted “a lot” as felt that it was subjective and difficult to quantify. Also felt that a child sweating at night is unusual therefore does not need to be “a lot”	How often does your child sweat while asleep?
Does your child wake up during night? (More than a child of a similar age) (27)	0.73	Added “how often.” Felt that the wording of this question had greater clarity (92.3%) than question 24. Although experts felt that there were many causes for night wakening and there is great variability between young children	How often does your child wake up during night? (More than a child of a similar age)
How often has your child had difficulty waking up in the morning? (28)	0.73	Added “even after getting plenty of sleep” and additional question; “How often is your child grumpy first thing in the morning?”	How often has your child had difficulty waking up in the morning even after getting plenty of sleep?
			How often is your child grumpy first thing in the morning?
Is your child unusually sleepy during the daytime? (31)	0.86	Added “how often” to fit response format and changed “daytime” to “day”	How often is your child unusually sleepy during the day?
Does your child appear to be more hyperactive than children of a similar age?	0.79	Added “how often” to fit response format and “fidgety” to add extra description to hyperactive	How often does your child appear more hyperactive or fidgety than children of a similar age?

The data on clarity and appropriateness were also used to contribute to decisions about changes needed and whether or not a question was retained.

Of the finally selected questions, four were modified from Gozal’s adaptation of Brouillettes questionnaire, three items were adaptation of the SDIS-C, two items were sourced from the OSA-18, one item was from the PSQ, and four items were new questions. None were chosen from the HK-CSQ, which may reflect the fact that this was developed for an older age group. The fact that questions retained came from a number of different sources suggests no systematic selection bias.

### Cognitive Interviews

The two parents interviewed had young children with DS previously investigated for OSA. They were members of a local DS support group and were health professionals. They suggested revision of the phrasing of 3 of the 14 selected questions as well as improvements to the instructions at the beginning of the questionnaire (see Table 3). Importantly, they felt that a typical week would be easier to recall than the last typical week in the last 6 weeks. They also recommended extending the number of options in the response format.

**Table 3:** **Modifications made to questionnaire after cognitive interview with parents**.

Questionnaire item	Final version
How often do you nudge/touch your child to make them breathe again when asleep?	When your child is asleep, how often do you nudge/touch your child to make them breathe again?
How often does your child sleep in strange positions such as tilting the head backwards or sleeping while sitting upright on pillows or kneeling?	How often does your child sleep in unusual positions? Examples of this are: tilting the head backwards; sleeping while sitting upright; kneeling with their bottom in the air
How often has your child had difficulty waking up in the morning even after getting plenty of sleep?	How often does your child have difficulty waking up in the morning, even after getting plenty of sleep?
The response format for the first draft of the questionnaire was: *Never, Rarely, Occasionally, Almost Always, or Unsure*	Never (never in the past 6 months) Rarely (<1 night a week) Occasionally (1–3 nights a week) Almost always (4–6 nights a week) Always (every night) Unsure
Changes to instructions and layout	Please try to think of a typical week when your child has been well Please **circle** the response you feel is most appropriate for your child from the options Never, rarely, occasionally, almost always, always or unsure Please only select **one** option for each of the questions and please answer **all** the questions

*This section of the table shows the modifications made to instructions at the beginning of the questionnaire after cognitive interview. Parts of the instructions are highlighted in bold to emphasise them*.

As a consequence of the cognitive interviews, changes to the instructions were made, the phrasing of some questions was modified to improve readability and “always” was added to the response format.

### Readability

The Flesch Reading Ease score for the final question selection was appropriate at 66.

## Discussion

This is the first parent reported questionnaire designed to screen young children with DS for obstructive sleep apnea. Using questions from previously validated questionnaires developed for other populations, we have used a structured approach involving both health professionals and parents of children with DS to adapt items and construct the new questionnaire.

Using an expert panel of relevant health professionals from a range of backgrounds and disciplines enabled a transparent process of assessment of items. The structured format in which the potential questions were presented to experts, enabled views to be obtained in a rigorous manner. Specifically, it enabled comment to be made on item relevance, clarity, and age appropriateness as well as content coverage of the items combined.

We included between two and eight items for each symptom for consideration by the expert panel, which enabled the best items to be selected for relevance. Children with DS have other medical problems, which may present with symptoms similar to those attributed to OSA in otherwise healthy children with normal development. This was reflected in the low rating for relevance of some items included in other questionnaires such as “Has your child stopped growing at a normal rate since birth?” Although growth restriction may result from OSA in children with DS, parents may interpret this question as referring to the different growth pattern of a child with DS, compared to their normally developing peers.

However, another example where similar concerns might apply, is “How often does your child appear more hyperactive or fidgety than children of a similar age?” This was rated relevant by the experts, emphasizing the importance of a range of professional views rather than simply relying on a small group of researchers who may have a biased view.

User input into questionnaire design is important in maximizing face validity. The views of parents in this study provided valuable additional feedback to improve clarity, and provided further evidence of this aspect of content validity.

### Limitations of the Study

Although expert panels allow for a wide range of opinion to be expressed and a level of agreement reached, there may be difficulties where some items receive CVI scores of less than the “acceptable” level of 0.78. It was felt necessary to include such items to ensure adequate content coverage such that questionnaire length was not unduly reduced prior to further psychometric testing. We have therefore included some questions for these reasons, which in their original wording, were not rated by all experts as highly relevant. Although the scale level CVI was adequate, this could be seen as weakening the case for content validity, but conversely, removing too many items too early in the development process is recognized as potentially problematic. A second round of content review could have been carried out by the expert group using the reworded questions in order to reassess the CVI, but, given that the items would still need to be tested more rigorously with psychometric analysis, this was not judged to be a useful exercise.

The parents who contributed to the cognitive interviews were active members of DS support groups, from healthcare backgrounds with a relatively high educational level and may not represent the whole population of parents looking after children with DS in the UK. Although their opinions and confidence in speaking aloud helped to modify the questions, different responses may have been obtained from using a wider group. Ideally, cognitive interviews would have been conducted with more families across a broader range of educational ability and cultural background. However, we were limited by the parents who volunteered to participate within the time period of the study. Nonetheless, the Flesch Reading Ease score shows that the language used should be accessible to parents of lower reading ability (31).

### Further Work

The items used in this questionnaire were taken from other validated scales. However, evidence for validity must be acquired afresh when the items are used in a different tool and for a different patient group to the original. Although we have started this process, work now needs to be done to provide evidence of construct validity, internal consistency, and test–retest reliability as well as sensitivity to change. This work has commenced with the use of the questionnaire in a large study of young children aged 6 months to 6 years with DS (30).

## Conclusion

Pediatric sleep questionnaires use parents’ observations of their child’s sleep to formulate a view of sleep behavior that can be used in a clinical context, in this case to screen for OSA in children with DS. Questionnaires should aid clinical decision making and reduce errors of judgment. In this context, the questionnaire should describe a symptom or behavior associated with OSA to the parent observer in a clear manner. This ensures the perception of a symptom or behavior between clinician and parent is the same. Where parents observe clinically significant signs in their DS children, such as apnea, they do not always report this to their child’s pediatrician (15), and similarly, physicians may overlook OSA in a child assessed during the daylight waking hours. The use of a questionnaire should therefore reduce bias and errors associated with human judgment (19). We hope to have achieved the first important step in the production of an unbiased questionnaire using a systematic approach to optimize content validity and clarity of presentation.

## Author Contributions

CH and HE conceived the idea for the study, CH, HE, and CT designed the work, and ES carried out the data collection. All authors were involved with data analysis and interpretation, all were involved with drafting and revising the paper, and all have approved the final draft.

## Conflict of Interest Statement

Dr. Catherine Mary Hill has received ­honoraria from Janssen Pharmaceuticals by way of speaker’s fees and a study grant from Respironics UK (2006) to attend an overseas course. The remaining co-authors declare that the research was conducted in the absence of any commercial or financial relationships that could be construed as a potential conflict of interest.

## Appendix

**Final Questionnaire**

**Sleep Questionnaire for Children with Down syndrome**

Please try to think of a typical week when your child has been well.

Please **circle** the response you feel is most appropriate for your child from the options;

Never, Rarely, Occasionally, Almost Always, Always or Unsure.

Please only select **one** option for each of the questions and please answer **all** the questions.

**Child’s name………………………………………………………………………………**

**Child’s age………………… years** ……………….months

**Date completed questionnaire………/………/………**.

**Table:**

1	How often does your child snore when they do NOT have a cold?	**Never** (never in the past 6 months)	**Rarely** (less than one night a week)	**Occasionally** (1–3 nights a week)	**Almost always** (4–6 nights a week)	**Always** (every night)	**Unsure**
2	How often can you hear your child snoring from outside of the bedroom?	**Never** (never in the past 6 months)	**Rarely** (less than one night a week)	**Occasionally** (1–3 nights a week)	**Almost always** (4–6 nights a week)	**Always** (every night)	**Unsure**
3	How often does your child struggle to breathe while asleep?	**Never** (never in the past 6 months)	**Rarely** (less than one night a week)	**Occasionally** (1–3 nights a week)	**Almost always** (4–6 nights a week)	**Always** (every night)	**Unsure**
4	How often does your child’s breathing go quiet and then he/she gasps?	**Never** (never in the past 6 months)	**Rarely** (less than one night a week)	**Occasionally** (1–3 nights a week)	**Almost always** (4–6 nights a week)	**Always** (every night)	**Unsure**
5	When your child is asleep, how often do you nudge/touch your child to make them breathe again?	**Never** (never in the past 6 months)	**Rarely** (less than one night a week)	**Occasionally** (1–3 nights a week)	**Almost always** (4–6 nights a week)	**Always** (every night)	**Unsure**
6	How often does your child sleep in unusual positions? Examples of this are; • tilting the head backwards • sleeping while sitting upright • kneeling with their bottom in the air	**Never** (never in the past 6 months)	**Rarely** (less than one night a week)	**Occasionally** (1–3 nights a week)	**Almost always** (4–6 nights a week)	**Always** (every night)	**Unsure**
7	How often does your child have restless sleep?	**Never** (never in the past 6 months)	**Rarely** (less than one night a week)	**Occasionally** (1–3 nights a week)	**Almost always** (4–6 nights a week)	**Always** (every night)	**Unsure**
8	How often does your child sweat while asleep?	**Never** (never in the past 6 months)	**Rarely** (less than one night a week)	**Occasionally** (1–3 nights a week)	**Almost always** (4–6 nights a week)	**Always** (Every night)	**Unsure**
9	How often does your child wake up during night? (More than other children of a similar age)	**Never** (never in the past 6 months)	**Rarely** (less than one night a week)	**Occasionally** (1–3 nights a week)	**Almost always** (4–6 nights a week)	**Always** (every night)	**Unsure**
10	How often does your child have difficulty waking up in the morning, even after getting plenty of sleep?	**Never** (never in the past 6 months)	**Rarely** (less than one night a week)	**Occasionally** (1–3 nights a week)	**Almost always** (4–6 nights a week)	**Always** (every night)	**Unsure**
11	How often is your child grumpy first thing in the morning?	**Never** (never in the past 6 months)	**Rarely** (less than one night a week)	**Occasionally** (1–3 nights a week)	**Almost always** (4–6 nights a week)	**Always** (every night)	**Unsure**
12	How often does your child tend to breathe through their mouth during the day?	**Never** (never in the past 6 months)	**Rarely** (less than one night a week)	**Occasionally** (1–3 nights a week)	**Almost always** (4–6 nights a week)	**Always** (every night)	**Unsure**
13	How often is your child unusually sleepy during the day?	**Never** (never in the past 6 months)	**Rarely** (less than one night a week)	**Occasionally** (1–3 nights a week)	**Almost always** (4–6 nights a week)	**Always** (every night)	**Unsure**
14	How often does your child appear more hyperactive or fidgety than children of a similar age?	**Never** (never in the past 6 months)	**Rarely** (less than one night a week)	**Occasionally** (1–3 nights a week)	**Almost always** (4–6 nights a week)	**Always** (every night)	**Unsure**

## References

[1] Primhak R O’Brien C Sleep apnoea. Arch Dis Child Educ Pract Ed (2005) 90:87–91. 10.1136/adc.2005.072975

[2] Marcus CL Sleep-disordered breathing in children. Am J Respir Crit Care Med (2001) 164:16–30. 10.1164/ajrccm.164.1.2008171 11435234

[3] de Miguel-Diez J Villa-Asensi JR Alvarez-Sala JL. Prevalence of sleep-disordered breathing in children with Down syndrome: polygraphic findings in 108 children. Sleep (2003) 26:1006–9. 14746382 10.1093/sleep/26.8.1006

[4] Marcus CL Keens TG Bautista DB von Pechmann WS Ward SL. Obstructive sleep apnea in children with Down syndrome. Pediatrics (1991) 88:132. 1829151

[5] Stores RJ Stores G. The significance of aspects of screening for obstructive sleep apnoea in children with Down syndrome. J Intellect Disabil Res (2014) 58(4):381–92. 10.1111/jir.12033 23489956

[6] Owens JA. Neurocognitive and behavioural impact of sleep disordered breathing in children. Pediatr Pulmonol (2009) 44:417–22. 10.1002/ppul.20981 19382210

[7] Churchill SS Kieckhefer GM Bjornson KF Herting JR. Relationship between sleep disturbance and functional outcomes in daily life habits of children with Down syndrome. Sleep (2015) 38(1):61–71. 10.5665/sleep.4326 25325444 PMC4262957

[8] Breslin J Spano G Bootzin R Anand P Nadel L Edgin J. Obstructive sleep apnea syndrome and cognition in Down syndrome. Dev Med Child Neurol (2014) 56:657–64. 10.1111/dmcn.12376 24471822

[9] Marcus CL Moore RH Rosen CL Giordani B Garetz SL Taylor HG A randomized trial of adenotonsillectomy for childhood sleep apnea. N Engl J Med (2013) 368:2366–76. 10.1056/NEJMoa1215881 23692173 PMC3756808

[10] Ng DK Chan CH Cheung JM Children with Down syndrome and OSA do not necessarily snore. Arch Dis Child (2007) 92:1047–8. 17954492 PMC2083588

[11] Dyken ME Lin-Dyken DC Poulton S Zimmerman MB Sedars E. Prospective polysomnographic analysis of obstructive sleep apnea in Down syndrome. Arch Pediatr Adolesc Med (2003) 157:655–60. 10.1001/archpedi.157.7.655 12860786

[12] Sproson EL Hogan AM Hill CM. Accuracy of clinical assessment of paediatric obstructive sleep apnoea in two English centres. J Laryngol Otol (2009) 22:1–8. 10.1017/S0022215109005532 19460184

[13] Brietzke SE Katz ES Roberson DW. Can history and physical examination reliably diagnose pediatric obstructive sleep apnea/hypopnea syndrome? A systematic review of the literature. Otolaryngol Head Neck Surg (2004) 131:827–32. 10.1016/j.otohns.2004.07.002 15577775

[14] Carroll JL McColley SA Marcus CL Curtis S Loughlin GM. Inability of clinical history to distinguish primary snoring from obstructive sleep apnea syndrome in children. Chest (1995) 108:610–8. 10.1378/chest.108.3.610 7656605

[15] Lin SC Davey MJ Horne RS Nixon GM. Screening for obstructive sleep apnea in children with Down syndrome. J Pediatr (2014) 165:177–122. 10.1016/j.jpeds.2014.02.032 24679609

[16] Rosen D Lombardo A Skotko B Davidson EJ. Parental perceptions of sleep disturbances and sleep-disordered breathing in children with Down syndrome. Clin Pediatr (Phila) (2011) 50:121–5. 10.1177/0009922810384260 21098528 PMC3348945

[17] Bull MJ. Health supervision for children with Down syndrome. Pediatrics (2011) 128:393–406. 10.1542/peds.2011-1605 21788214

[18] Royal College of Paediatrics and Child Health. Working Party on Sleep Physiology and Respiratory Control Disorders in Childhood, Standards for Services for Children with Disorders of Sleep Physiology Report (2009). pp 23–33.

[19] Spruyt K Gozal D. Paediatric Sleep Questionnaires as diagnostic or epidemiological tools: a review of currently available instruments. Sleep Med Rev (2011) 15:19–32. 10.1016/j.smrv.2010.07.005 20934896 PMC3088759

[20] Chervin RD Hedger K Dillon JE Pituch KJ. Pediatric Sleep Questionnaire (PSQ): validity and reliability of scales for sleep-disordered breathing, snoring, sleepiness, and behavioral problems. Sleep Med (2000) 1:21–32. 10.1016/S1389-9457(99)00009-X 10733617

[21] Spruyt K Gozal D. Development of pediatric sleep questionnaires as diagnostic tools: a brief review of dos and don’ts. Sleep Med Rev (2011) 15:7–17. 10.1016/j.smrv.2010.06.003 20952230 PMC3022091

[22] Slocumb EM Cole FLA. Practical approach to content validation. Appl Nurs Res (1991) 4:192–5. 10.1016/S0897-1897(05)80097-7 1772253

[23] Constantin E Tewfik T Brouillette R. Can the OSA-18 quality-of-life questionnaire detect obstructive sleep apnea in children? Pediatrics (2010) 125:e162–8. 10.1542/peds.2009-0731 20026494

[24] Luginbuehl M Bradley-Klug KL Ferron J Anderson WM Benbadis SR. Pediatric sleep disorders: validation of the sleep disorders inventory for students. School Psych Rev (2008) 37:409–31. 10.1016/j.sleep.2014.03.021 24954442

[25] Gozal D. Sleep-disordered breathing and school performance in children. Pediatrics (1998) 102:616–20. 10.1542/peds.102.3.616 9738185

[26] Li AM Cheung A Chan D Wong E Ho C Lau J Validation of a questionnaire instrument for prediction of obstructive sleep apnea in Hong Kong Chinese children. Pediatr Pulmonol (2006) 41:1153–60. 10.1002/ppul.20505 17054110

[27] Lynn MR Determination and quantification of content validity. Nurs Res (1986) 35:382–6. 10.1097/00006199-198611000-00017 3640358

[28] Polit DF Beck CT Owen SV. Is the CVI an acceptable indicator of content validity? Appraisal and recommendations. Res Nurs Health (2007) 30:459–67. 10.1002/nur.20199 17654487

[29] Willis G Cognitive Interviews – A Tool for Improving Questionnaire Design. Thousand Oaks, CA: SAGE (2005).

[30] Hill CM Ashworth A Elphick H Evans H Farquhar M Gavlak J Prevalence of obstructive sleep apnoea in young children with Down syndrome: a domiciliary cardiorespiratory study. Abstract, Biennial Meeting of the European Sleep Research Society, Tallinn, Estonia, 16-20 September (2014).

[31] Flesch R A new readability yardstick. J Appl Psychol (1948) 32:221–33. 10.1037/h0057532 18867058

